# Unilateral Biportal Endoscopic (UBE) Treatment of Symptomatic Lumbar Facet Cysts After Interspinous Ligamentoplasty: Report of Two Cases and Literature Review

**DOI:** 10.7759/cureus.90663

**Published:** 2025-08-21

**Authors:** Kang Suk Moon, Pedro Leonardo Villanueva-Solórzano, Michel G Mondragón-Soto, Chungnam Lee, Hyun-Nam Seo

**Affiliations:** 1 Department of Spine Surgery, Ileona Hospital, Siheung, KOR

**Keywords:** degenerative lumbar spondylolisthesis, facet cysts, interspinous ligamentoplasty, lumbar spine stenosis, minimally invasive spine surgery, unilateral biportal endoscopic

## Abstract

Interspinous ligamentoplasty (ILP) is an effective, minimally invasive technique used to treat low-grade degenerative spondylolisthesis (DSL) and lumbar spinal stenosis (LSS). However, it may be associated with postoperative complications, including the formation of a facet cyst (FC). Various surgical strategies have been developed to address these clinical challenges. In recent decades, minimally invasive techniques, such as unilateral biportal endoscopy (UBE), have gained popularity for achieving adequate decompression while preserving segmental stability. We report two cases of patients with Grade I DSL and LSS who initially experienced symptomatic relief following treatment with ILP. Within months, both developed recurrent radicular pain and neurogenic claudication. Magnetic resonance imaging (MRI) revealed FCs at the previously decompressed levels. After the failure of conservative treatment, UBE-assisted cyst resection and decompression were performed. In both cases, operative time was brief, and patients achieved significant symptom resolution without perioperative complications. ILP may be a motion-preserving alternative to pedicle-based fusion for mild-to-moderate LSS with low-grade DSL. However, residual micromotion and facet stress may predispose to FC formation. UBE is a minimally invasive management alternative for cyst resection and nerve decompression, preserving facet integrity and potentially reducing the risk of adjacent segment disease. In patients with advanced instability, fusion may still be required. While early outcomes have shown to be favorable, the limited follow-up highlights the need for longitudinal studies to clarify long-term recurrence rates, indications for secondary fusion, and comparative outcomes against traditional fusion techniques. UBE may enable precise FC removal with minimal tissue disruption, offering an alternative option for recurrent radiculopathy after ILP. This approach may help preserve spinal motion while effectively addressing symptomatic lesions. Larger prospective studies with more extended follow-up periods are necessary to confirm these findings and refine treatment algorithms for FCs arising after ILP.

## Introduction

Lumbar spinal stenosis (LSS) and low-grade degenerative spondylolisthesis (DSL), which often coexist, represent two of the most prevalent causes of back pain, radiculopathy, and neurogenic claudication worldwide [1,2]. Symptoms typically progress insidiously until they become disabling, making these conditions among the most common indications for spine surgery in the elderly population [3]. Surgical strategies primarily involve direct decompression, such as laminectomy, with or without instrumented fusion, depending on the severity of DSL [4]. Although fusion aims to restore spinal stability, it entails risks of complications, including adjacent segment disease, pseudoarthrosis, and increased perioperative morbidity, particularly in older patients [5].

Interspinous ligamentoplasty (ILP), previously referred to as interspinous locker fixation (ILF), combines spinal decompression with the placement of a synthetic ligament, often composed of polyester fibers, wrapped around adjacent spinous processes to function as a posterior tension band. Some authors advocate the use of a titanium spacer, or "Locker", placed between spinous processes to enhance stability, although its use varies depending on surgeon preference [6,7].

ILP primarily targets low-grade DSL (Grade I), where micro-instability exists but does not necessitate rigid fixation with pedicle screws [6]. It is also suitable for patients with mild-to-moderate LSS, particularly those who are poor candidates for pedicle-based fusion due to high surgical risk, comorbidities, or advanced age [3,8]. However, higher-grade spondylolisthesis and markedly unstable segments typically require more rigid constructs [9-11]. Unlike rigid pedicle-based constructs, ILP employs a dynamic stabilization technique that achieves mild decompression of the spinous processes, unloads the facet joints, and potentially widens the foramen [12], dampening hypermobility in flexion and extension while preserving segmental motion, particularly axial rotation [6,13,14]. This approach may lower the risk of developing adjacent segment disease while restricting excessive flexion-extension movements to maintain a physiological range of motion and prevent overt spinal instability [12,15].

Over the past decade, numerous interspinous process devices (IPDs) have emerged as alternative motion-preserving technologies to ILP [15]. However, significant differences in design, material composition, and fixation mechanisms make direct comparisons of clinical outcomes and safety profiles challenging, as each device exerts a unique biomechanical effect on segment motion [16].

Despite its widespread use, data regarding long-term complication rates associated with ILP remain limited. Reported hardware-related issues, such as spinous process fractures, device loosening, or migration [8,17], may require revision surgery and contribute to the relatively high reoperation rates [1,8,18]. Persistent or recurrent symptoms may result from an inadequate decompression or unaddressed instability at the treated segment [8]. Progression of LSS may also occur in cases of insufficient segmental fixation [6,8]. Additionally, facet joint degenerative changes, including the potential formation of synovial cysts, have also been reported as a potential sequela [19,20].

Facet cysts (FCs) are synovial outpouchings of the facet joint capsule that may encroach upon the spinal canal or neural foramina [21,22]. They commonly arise in the setting of facet arthropathy or segmental instability [23,24]. Rigid fusion with motion restriction significantly reduces the likelihood of FC formation at the treated level [25,26]. In contrast, spinous process-based fusions or dynamic fixations may permit persistent micromotion, predisposing to FC formation or enlargement, a rare but acknowledged complication [19,20,27]. When FCs compress adjacent nerve roots, patients may present with local pain, radiculopathy, weakness, neurogenic claudication, or sensory deficits [21]. In patients previously treated with either ILP or fusion procedures, postoperative radicular pain should prompt the suspicion of residual or recurrent stenosis, implant-related issues (e.g., migration or malposition), or new FC development secondary to altered segmental biomechanics [17,18,20,28].

Due to the variability in devices, patient selection criteria, and surgical technique, questions persist regarding the safety profile of ILP, particularly concerning FC formation resulting from residual micromotion [2,8,18,27]. Although some studies have documented de novo formation of FC after decompression or posterior instrumentation, few specifically address cysts arising after ILP [14,22]. Evidence is thus extrapolated from other clinical scenarios, highlighting a persistent knowledge gap regarding the true incidence, risk factors, and optimal management strategies for this complication [29].

This manuscript underscores the importance of minimally invasive spine surgery, particularly endoscopic techniques, including unilateral biportal endoscopy (UBE) for the management of postoperative FCs. This approach offers a fusion-free alternative that may achieve favorable clinical outcomes while preserving spinal motion and reducing procedural morbidity.

## Case presentation

Case 1

A 66-year-old female with a history of ILP and decompression at the L3-L4 level six months prior presented with left-sided sacral (Visual Analog Scale (VAS) 8) and leg pain (VAS 8) accompanied by neurogenic claudication and left lower limb weakness (Grade 4-/5) [30]. A diagnostic left L3 nerve root block resulted in temporary symptom relief (VAS 4), but her symptoms recurred shortly thereafter. MRI showed a left-sided FC at the L3-4 level causing nerve root compression. Given her symptoms and failure of conservative measures, surgical intervention was deemed necessary.

**Figure 1:**
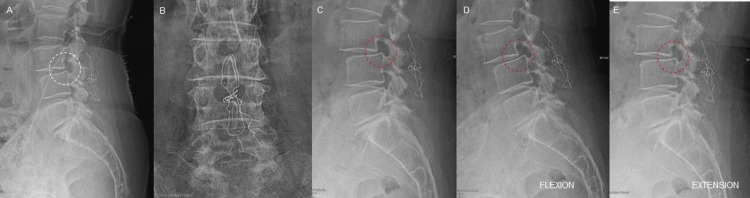
Case 1 Progression of Slippage X-Rays Case 1: Post-ILP radiographs lateral (A) and AP (B) views taken two months after the first surgery showed preserved segmental motion and minimal translation at the L3-L4 level (A, white circle). Six months postoperative lateral radiographs in neutral (C), flexion (D), and extension (E) position showing slippage progression and dynamic angular instability secondary to the spondylolisthesis at the previously mentioned level (encompassed in the red circle). ILP: interspinous ligamentoplasty, AP: anteroposterior.

**Figure 2:**
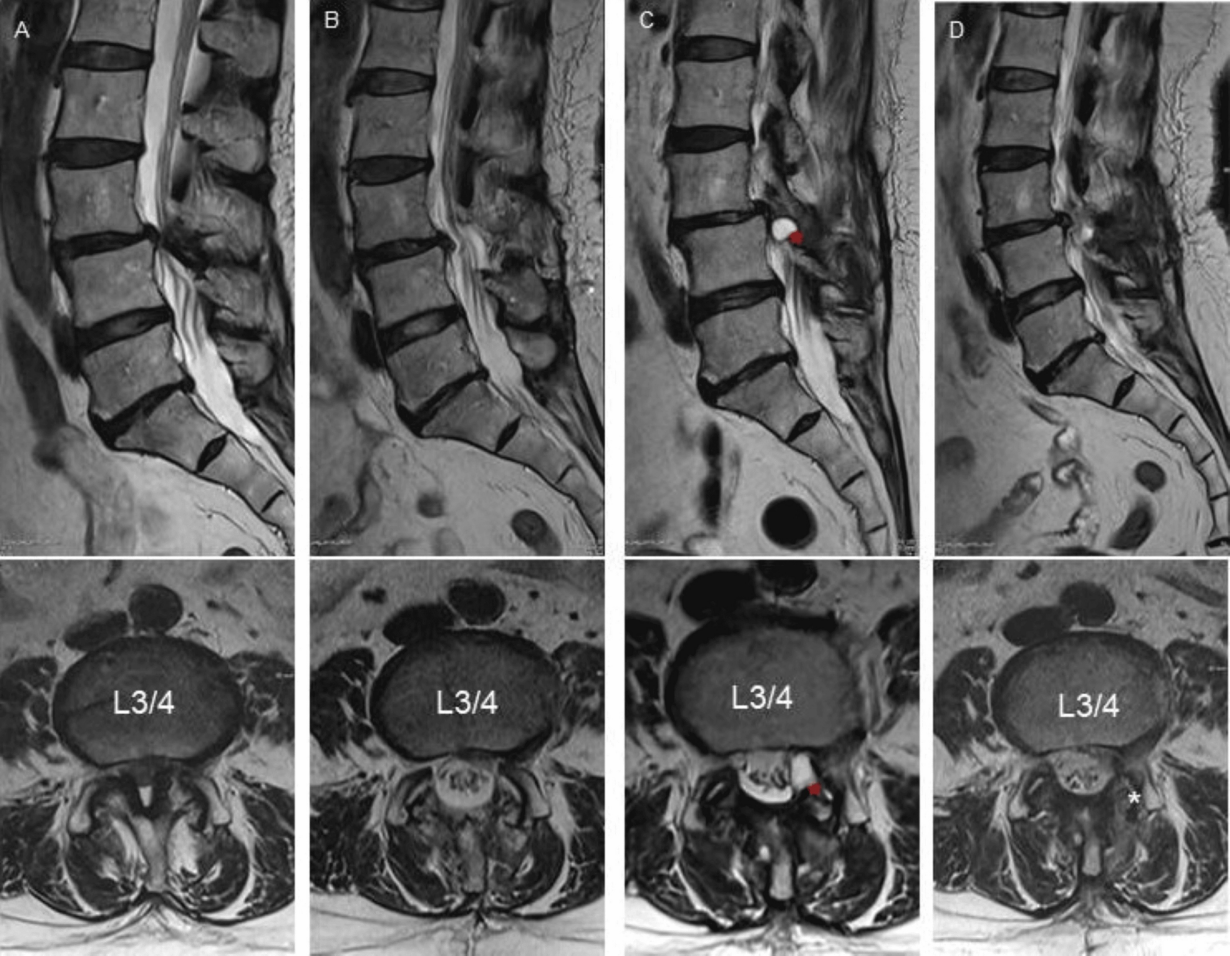
Case 1 MRI Progression Timeline Sagittal and axial T2-weighted MRI. Preoperative images (L3-4 LSS + DSL Grade 1) (A). Post-ILP decompression state (B). FC (red asterisk *) development and lateral recess stenosis (C). Post-FC resection and lateral recess decompression state (D). LSS: lumbar spinal stenosis, DSL: degenerative spondylolisthesis, ILP: interspinous ligamentoplasty, FC: facet cyst.

The patient underwent a UBE surgery with left-sided laminectomy at the L3-4 level to decompress the affected nerve root. The total operative time was 40 minutes. Her symptoms resolved (VAS 1 for lumbar pain and VAS 0 for radicular pain) with no further complications. Her strength improved to full power a few weeks after surgery (Figures 1A-1E, 2A-2D).

Case 2

A 48-year-old male with a past medical history of ILP and decompression at L3-5 levels seven months prior presented with persistent sacral pain, progressive lower back discomfort (VAS 6), and left lower limb radicular symptoms (VAS 8). MRI revealed an FC at L3-4 and a left foraminal disc herniation at L4-5. A diagnostic left L4 nerve root block provided transient relief (VAS 3), but symptoms progressed despite conservative management.

**Figure 3:**
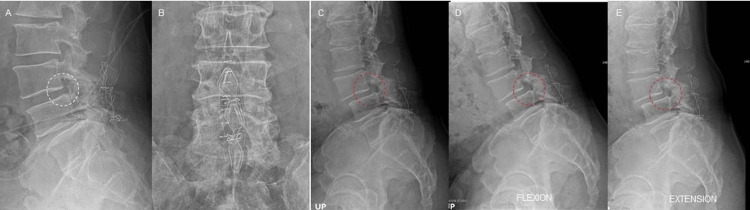
Case 2 Progression of Slippage Postoperative ILP X-ray lateral (A) and AP (B) lumbar X-ray views taken two months after the first surgery, showing the artificial ligament placed around spinous processes with preserved segmental motion and minimal translation (A, white circle). Seven months postoperative dynamic views (C-E) showing progression of slippage (red circle). ILP: interspinous ligamentoplasty, AP: anteroposterior.

**Figure 4:**
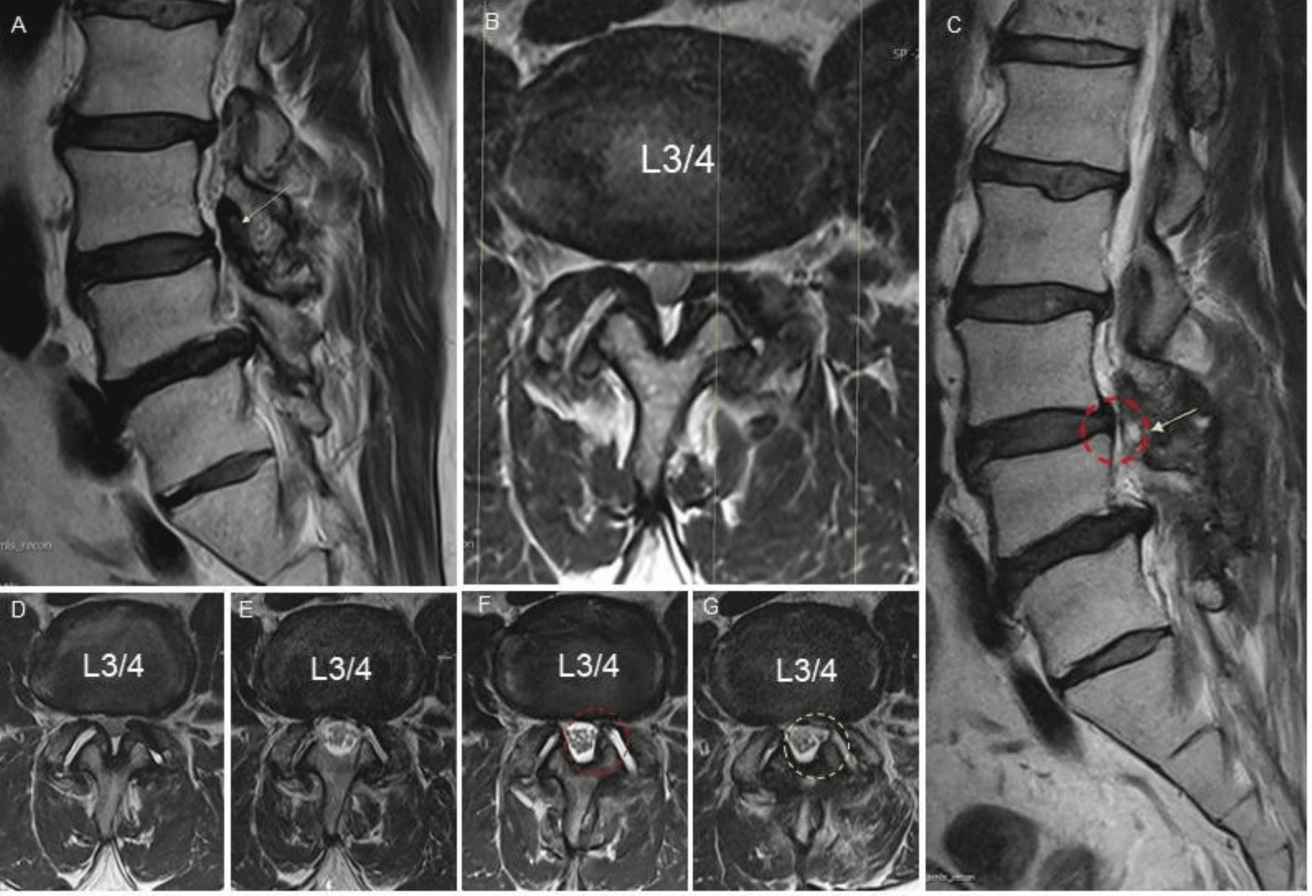
Case 2 MRI Timeline Sagittal and axial T2-weighted MRI preoperative images (L3-4 LSS + DSL Grade 1 and L4-5 foraminal stenosis) (white arrow indicating the L3-4 level) (A) with facet degeneration (B). Sagittal view of the FC lesion location (red circle) after seven months period follow-up (C). Progression timeline axial T2-weighted MRIs: Preoperative (D), Post-ILP (E), FC development (F), and final decompressed lateral recess state (white circle) (G). LSS: lumbar spinal stenosis, DSL: degenerative spondylolisthesis, ILP: interspinous ligamentoplasty, FC: facet cyst.

Revision UBE surgery was performed, consisting of a left-sided decompression at L3-4 to address the FC, and a paraspinal approach at L4/5 to decompress the foramen and exiting nerve root. The operative time for the whole procedure was 120 minutes. The patient experienced significant symptom relief (VAS 1 for back pain and VAS 1 for radicular pain) and improved mobility postoperatively (Figures 3A-3E, 4A-4G).

Surgical technique description

The patient was positioned prone under general anesthesia. A UBE procedure was performed at the target level under fluoroscopic guidance to ensure accurate localization. Two small incisions were made: one for insertion of the endoscope, arthroscopic sleeve, and irrigation system, and the other for the introduction of surgical instruments and outflow of saline irrigation.

**Figure 5:**
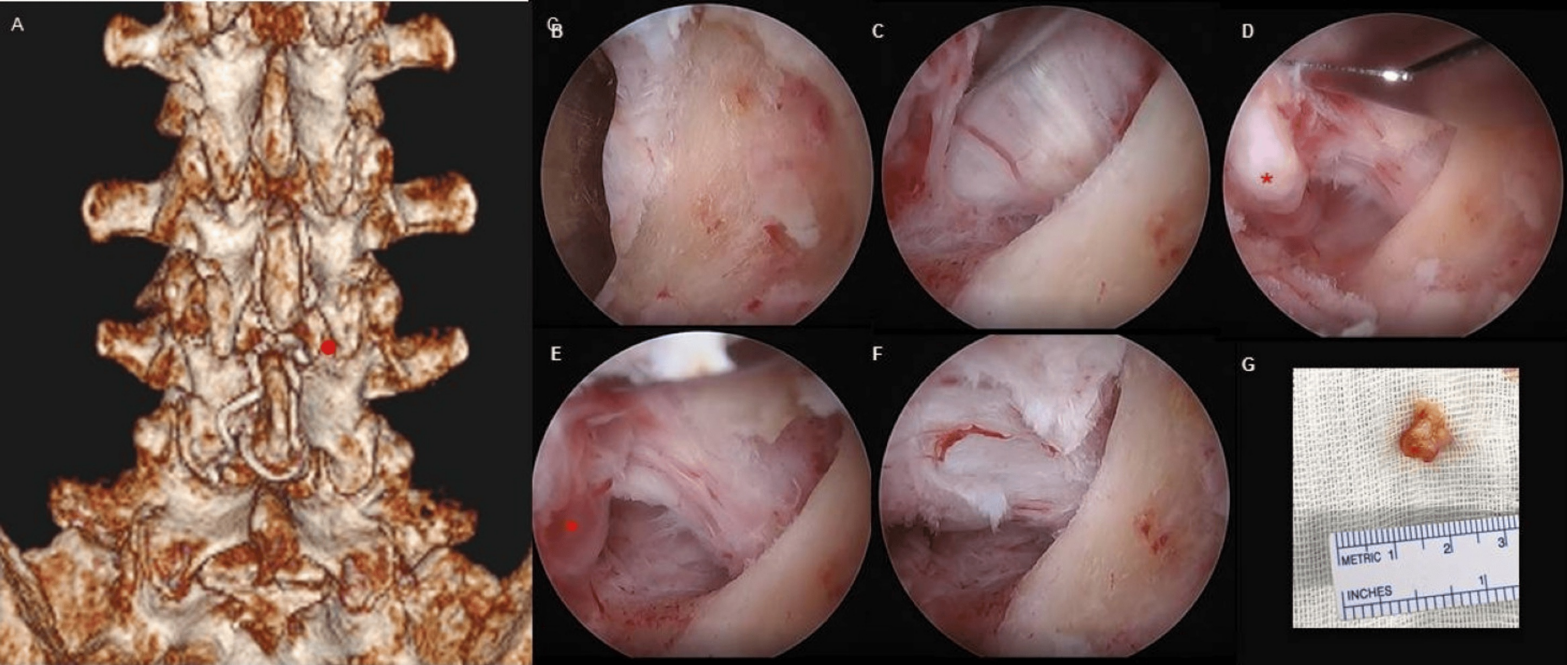
UBE Target Location at the Lateral Recess Junction Zone CT reconstruction of the lumbar spine of a patient with a previous ILP placement. The red dot indicates the location corresponding to the right lateral recess (A). Intraoperative images L3-4 level and endoscopic technique sequence for the FC identification and resection;  initial target area location (B), clear cleavage plane preparation between the lesion and the dura (C), FC (red asterisk *) with typical outpouch appearance arising from the joint capsule with surrounding adhesions (D), traversing nerve root widely exposed with surrounding structures visualized (red dot) (E), final decompression state (F), and macroscopic appearance of the resected FC (G). UBE: unilateral biportal endoscopy, ILP: interspinous ligamentoplasty, FC: facet cyst.

The procedure began with minimal drilling of the ipsilateral facet joint and lamina to allow adequate access. Following this, the FC was noticeably identified, and a clean dissection plane was developed between the lesion and the dura. Upon full exposure, the FC was carefully drained and resected. Endoscopic images (Figures 5A-5G) illustrate the FC and its anatomical relationship to the thecal sac and the traversing nerve root. The procedure concluded with endoscopic confirmation of adequate nerve root decompression. The portals were closed using 3-0 nylon sutures. This surgical technique was employed in both cases described above.

## Discussion

Both patients initially experienced disabling LSS and grade I DSL and underwent ILP, achieving satisfactory early outcomes. However, symptoms recurred within months due to the formation of FC at the previously decompressed levels. This troublesome complication is caused by persistent instability and facet joint stress, which can foster lesion formation even after initial decompression, and it represents a clinical and therapeutic challenge. Post-surgical FC formation rates are approximately 15%, particularly in segments with mild DSL. Failure of conservative management often necessitates revision surgery [31].

Dynamic constructs, such as ILP, maintain segmental motion, which may potentially reduce the risk of adjacent segment disease. However, micromotion may perpetuate facet joint stress, encouraging synovial FC formation [7]. Conversely, fusion surgeries reduce the risk of such complications, but they carry risks of increased morbidity and adjacent segment disease, favoring motion-preserving alternatives in selected cases [15,26,28].

As mentioned above, both ILP and fusion eliminate the motion at the treated level, which in succession transfers mechanical loads and motion to the adjacent segments, which is the hallmark of adjacent segment disease (ASD). These circumstances lead to accelerated degeneration of the facets and discs juxtaposed to a fusion, which in turn predisposes to facet joint arthritis and instability [10]. Continued joint deterioration perpetuates inflammatory changes, micro-instability, and joint effusion, which can force fluid out of the weakened joint capsule, dilating the latter and forming a cystic protrusion [32]; to a much lesser extent, FCs have been reported to arise within the ligamentum flavum and interspinous ligaments [9]. These FCs are characterized by having a synovial lining [33], which histologically has a lining of synovial cells, and frequently appear with signs of chronic inflammation or degeneration, such as fibroblast proliferation, myxoid degeneration of collagen, and high hyaluronic acid content [34]. In already affected spine levels, segmental instability and minor trauma may play a significant role in the FC formation [35]. The categorical association of FCs with osteoarthritis (40.5%), spondylolisthesis (43.4%), and disc degeneration (13.2%) underscores their degenerative essence [34,36].

Clinical management should be tailored according to the symptomatology of the patients, which often correlates with the severity of the radiological findings. Other essential factors that should be considered include the surgeon’s expertise and the patient’s preferences. Current treatment modalities include conservative (percutaneous cyst aspiration and steroid injections) and surgical management, which is often preferred after the conservative management has failed [37-39].

Magnetic resonance imaging (MRI) remains the gold standard for diagnosing facet cysts, which appear as hyperintense lesions on T2-weighted images adjacent to the facet joint [31]. Selective nerve root or facet joint blocks may serve for both diagnostic and therapeutic purposes; temporary pain relief after injection suggests that the facet cyst is the pain generator, thus aiding in the decision for surgical management. For patients with FCs with no clear clinical and radiological evidence of segmental instability, minimally invasive approaches without fusion may offer the best surgical outcomes, achieving symptomatic relief with an acceptable safety profile, and maintaining segmental stability by avoiding excessive facet joint sacrifice [39].

The surgical options currently available include simple decompression with FC resection to fusion extension. However, in recent years, the total cyst excision via a small flavectomy has been considered the surgical therapy of choice as it represents the least invasive approach [40]. Surgical approaches include traditional percutaneous approaches (hemilaminectomy or bilateral laminectomy) and minimally invasive procedures (tubular retractors or endoscopic) [40].

UBE is a minimally invasive, facet-preserving alternative for managing symptomatic FC and underlying stenosis. By employing two small working portals, the surgeon achieves a high-resolution, magnified view of the pathology. This approach facilitates precise cyst removal and decompression while preserving critical structures such as facet joints, ligamentous attachments, and paraspinal musculature. The enhanced endoscopic field aids in identifying cyst margins and addressing dural adhesions, ensuring complete FC excision without leaving remnants, which might trigger recurrence. This technique addresses both FC removal and stenosis with minimal bony resection and soft tissue disruption, reducing the risk of inadvertent dural tears, symptomatic recurrence, and postoperative instability [1,2].

Additionally, UBE’s versatility allows simultaneous treatment of coexisting lumbar pathologies, such as lateral recess stenosis and disc herniations [4,5]. While other minimally invasive techniques may be able to satisfactorily resolve these shortcomings, recently the focus in our practice has extended into exploring endoscopic alternatives due to shortened operative time which minimizes anesthesthetic exposure, lower infection rate associated to constant saline flow in the surgical site, and other advantages such as water dissection of tight adhesions enabled by the solution inflow, and decreased need for multiple surgical interventions [8,12,37,39,40].

On the other hand, UBE has been associated with a steep learning curve, which may need time and practice to overcome, and this may imply that surgical time could be prolonged during the learning phase. Nonetheless, depending on surgeons preference and very specific technical requirements, such as the endoscope, the arthroscope and the irrigation system, other minimally invasive options may be opted for. Other shortcomings may be encountered include limited angle of vision for areas outside of the surgical objective, limited maneuverability, risk of epidural hematoma formation, and dural tear, which may be addressed endoscopically with a surgical hemostatic patch if the defect is small (<1 cm), or may require conversion into an open procedure if the defect is larger (>1 cm) for direct repair with suture or other strategies.

By achieving complete cyst removal and adequate decompression, UBE lowers the likelihood of symptomatic recurrence and adjacent segment disease, often eliminating the need for routine fusion or subsequent surgical interventions in appropriately selected cases [6,7,41]. In a systematic review, Benato et al. [26] reported that in six studies included in their research, encompassing 657 patients, comparing outcomes between lumbar posterior decompression and lumbar decompression and fusion, the latter group was found to be associated with better outcomes in terms of less postoperative lumbar pain and lower cyst recurrence rates than decompression alone; however, there were no differences in terms of complications, reintervention rates.

In a meta-analysis, Chen et al. [39] compare the clinical outcomes between minimally invasive approaches using tubular retractors (microscopic vs. endoscopic) and traditional percutaneous approaches for the treatment of FCs. A total of 1,833 patients from 41 studies and their series were considered, and they reported no statistically significant difference in pain management, dural tear, residual cyst, recurrence, and operation time between minimally invasive surgery groups and traditional surgical treatment groups. Nonetheless, minimal groups had optimized functional improvement and lower reoperation rates. Intraoperative bleeding and in-hospital stay were statistically significantly lower than those of the conventional group. In the subgroup analysis between the minimally invasive methods, endoscopic approaches had a shorter operating time, and there was no other significant difference in the rest of the variables studied.

Despite the well-defined benefits in radicular symptom relief, the underlying biomechanical milieu, characterized by micromotion, instability, and facet stress, may persist even after improvement secondary to the cyst removal [16,18,27]. In cases with significant instability, a secondary fusion procedure should be considered. Current improvement niches in FC management include the ability to predict postoperative recurrence. A work conducted on 89 patients implemented the Lumbar Cyst Score model, which proved to be a rapid and accurate tool in which the probability of FC recurrence after surgery predicted <5% for a score of 2 or less to >88% for a score of 7 [42].

The most recent research has centered on the use of artificial intelligence (AI) in the spine field, which has been increasingly reported in the last decade, with groundbreaking and disruptive applications [43]. These include localizations of specific radiological findings, classification and regression tasks focusing on diagnostic processes, and supportive tools to predict outcomes and anticipate the most advantageous treatment and prevention methods [44].

Further studies are needed to establish the long-term outcomes of UBE for facet cysts, to better understand and predict recurrence rates, and indications for additional fusion procedures, starting with longitudinal observational studies to determine risk factors associated with FCs. Posteriorly, randomized controlled trials with extended follow-up are necessary to validate these observations, refine patient selection criteria, and determine the long-term efficacy and safety of UBE resection for symptomatic facet cysts following ILP.

This manuscript is subject to the inherent limitations of a technical note and case report. The small number of patients and the absence of a control group limit the ability to draw broad conclusions or establish definitive treatment protocols. Furthermore, the short follow-up period restricts insights into long-term outcomes, including potential recurrence rates or progressive instability. As such, these findings may not be widely generalizable across diverse patient populations or clinical settings.

## Conclusions

ILP provides a motion-preserving alternative to pedicle-based fusion in low-grade DSL and mild-to-moderate LSS. However, residual micromotion and facet joint stress may contribute to the formation of FCs, leading to recurrent pain and neurological symptoms. UBE may be considered as a minimally invasive alternative that could enable precise cyst resection while preserving facet integrity and minimizing postoperative pain, blood loss, and recovery time. In the absence of significant instability, this may be sufficient to relieve symptoms, reserving fusion for cases with persistent or progressive instability. Overall, this strategy highlights the efficacy of minimally invasive techniques in achieving durable symptom relief while maintaining spinal stability.
